# A sustainable process for procuring biologically active fractions of high-purity xylooligosaccharides and water-soluble lignin from *Moso* bamboo prehydrolyzate

**DOI:** 10.1186/s13068-019-1527-3

**Published:** 2019-07-29

**Authors:** Caoxing Huang, Xucai Wang, Chen Liang, Xiao Jiang, Gan Yang, Jie Xu, Qiang Yong

**Affiliations:** 1 Jiangsu Co-Innovation Center for Efficient Processing and Utilization of Forest Resources, College of Chemical Engineering, Nanjing Forestry University, Longpan Road 159, Nanjing, 210037 China; 2 Guangxi Key Laboratory of Clean Pulp & Papermaking and Pollution Control, Nanning, 530004 China; 3 Department of Forest Biomaterials, North Carolina State University, Campus Box 8005, Raleigh, NC 27695-8005 USA

**Keywords:** Prehydrolyzate, Polystyrene divinylbenzene resin, Xylooligosaccharides, Soluble lignin, Biological active

## Abstract

**Background:**

Prehydrolyzate, which is from the prehydrolysis process in dissolving pulps industry, contains various sugar-derived and lignin compounds such as xylooligosaccharides (XOS), gluco-oligosaccharides, xylose, glucose, and soluble lignin (S-L). The XOS has several beneficial effects on human physiology. XOS and S-L in prehydrolyzate are difficult to efficiently fractionate due to their similar molecular weights and water solubility. In this work, we proposed a sustainable and green process using polystyrene divinylbenzene (PS-DVB) resin to simultaneously separate and recover XOS and S-L. Enzymatic hydrolysis with endo-1,4-β-xylanase and fermentation with *P. stipites* were sequentially applied to purify XOS to minimize xylose content as well as amplify contents of xylobiose and xylotriose. In addition, 2D-HSQC NMR was used to analyze the structural characteristics of XOS and S-L. Furthermore, the biological abilities of antioxidants and prebiotics of these fractions were investigated by scavenging radicals and cultivating intestinally beneficial bacterias, respectively.

**Results:**

Results showed that PS-DVB resin could simultaneously separate XOS and solubilized lignin with excellent yields of 93.2% and 85.3%, respectively. The obtained XOS after being purified by enzymatic hydrolysis and fermentation contained 57.7% of xylobiose and xylotriose. 10.4% amount of inherent xylan was found in the S-L fraction obtained by PS-DVB resin separation. 2D-HSQC NMR revealed that lignin carbohydrate complexes existed in both XOS and S-L as covalent linkages between lignin and 4-*O*-methylglucuronoarabinoxylan. The biological application results showed that the antioxidant capacity of S-L was stronger than XOS, while XOS was superior in promoting growth of intestinal *Bifidobacteria adolescentis* and stimulating production of short-chain fatty acids by *Lactobacillus acidophilus.*

**Conclusions:**

The proposed strategy of sequentially combining hydrophobic resin separation, enzymatic hydrolysis, and fermentation was successfully demonstrated and resulted in simultaneous production of high-quality XOS and solubilized lignin. These biomass-derived products in prehydrolyzate can be regarded as value-adding prebiotics and antioxidants.

**Electronic supplementary material:**

The online version of this article (10.1186/s13068-019-1527-3) contains supplementary material, which is available to authorized users.

## Background

Studies of world markets clearly indicate a trend of increasing demand for dissolving-grade pulps, a producible material from hardwood, softwood, bamboo, and cotton linters [1–3]. Currently, the process for the dissolving pulp comprises two distinct segments: the sulfite process and the prehydrolysis kraft process. For the latter process, water or steam prehydrolysis at 160–200 °C is carried out in a primary stage, resulting in partial hydrolysis of hemicelluloses and lignin degradation [4–6]. Constituents from biomass fractions can be dissolved in the prehydrolysis liquor, which is termed as prehydrolyzate. Prehydrolyzate contains various sugar-derived compounds such as xylooligosaccharides, gluco-oligosaccharides, xylose, and glucose [7]. In current practice process, the prehydrolyzate is mixed with black liquor from kraft cooking process and then burned to recover energy [8]. This current dispose does not account for the value of these biomass-derived products in prehydrolyzate; therefore, efficient recovery and utilization of valuable products should be realized.

Xylooligosaccharides (XOS) are defined as xylanic oligosaccharides that contain 2–10 xylose units linked together by β-1,4 glycosidic bonds. At present, XOS are commercially produced from xylan-rich lignocellulosic materials through the enzymatic hydrolysis using xylanase, dilute acid digestion using sulfuric acid or acetic acid, or a hot water pretreatment similar to previously described prehydrolysis [7–10]. Among these technologies, prehydrolysis is regarded as the most green process for producing XOS due to no chemical reagent added in the process. Based on this concept, it can be claimed that recovery of XOS from the prehydrolyzate generated during dissolving pulp industry appeals to the principles of “green chemistry” [11–13]. Biological function of XOS is most potent in samples with high purity, which indicates low contents of xylose and glucose alongside particular enrichment in xylobiose (X2) and xylotriose (X3). It is reported that employing a secondary enzymatic hydrolysis upon a XOS solution using endo-β-1-4-xylanase can degrade high-DP XOS to effectively reach X2 and X3 enrichment [14, 15]. Another means of purifying XOS is to use yeast to ferment the undesired monosaccharide impurities into ethanol, further elevating XOS purity [16]. Resultant ethanol can then be removed via distillation. Importantly, hot water pretreatment-derived XOS always contains undesired degradation products such as solubilized lignin, xylose, glucose. Hence, purification of XOS to remove low monosaccharides and lignin is an indispensable step in creating XOS products that can be eligible as food ingredients.

To isolate XOS from prehydrolyzate, technologies such as membrane ultrafiltration, activated carbon adsorption, and flocculation have been applied. Although ultrafiltration can separate the XOS with DP of 2–10 from prehydrolyzate containing higher molar mass XOS and lignin fragments, the solubilized lignin with molecular weights between ~ 1000 and 2000 g/mol can still contaminate the permeating XOS of interest [17, 18]. This mandates removal of solubilized lignin prior to ultrafiltration. In one report aiming to do this, Bing et al. [19] used polyaluminum chloride as a flocculant to remove the solubilized lignin in the prehydrolyzate. It was found that increasing flocculent dosage resulted in lignin removal from ~ 12 to 52%, yet the recovery yield of XOS problematically decreased from ~ 88 to 83% after lignin removal. In the work of Liu et al. [20], ~ 83% of solubilized lignin in the prehydrolyzate could be removed from prehydrolyzate using a mixture of polydimethyl diallyl ammonium chloride and activated carbon, yet only ~ 67% of the XOS was obtained afterward. These demonstrations show that it remains necessary to find an efficient and facile technology to simultaneously remove the solubilized lignin without hampering recovery of XOS. Polystyrene divinylbenzene resin (PS-DVB), a class of hydrophobically polymeric particles, can selectively adsorb hydrophobic aqueous-soluble molecules by *π*–*π*′ and van der Waal interactions [21, 22]. The adsorbed lignin in resin can then be desorbed by a mildly polar organic reagent such as ethanol and methanol [22]. Hence, we propose use of PS-DVB resin to separate the soluble lignin and XOS in the prehydrolyzate to ideally remove lignin contaminants without sacrificing XOS yield.

XOS is a kind of non-digestible oligosaccharide that has several beneficial effects on human physiology, such as reduced cholesterol, improve gastrointestinal health, reduce the risk of diabetes mellitus, and stimulate the growth of intestinal *Bifidobacteria* and *Lactobacillus acidophilus* [23, 24]. In addition, the XOS from biomass have shown the ability to scavenge free radicals [25]. The lignin has also been verified as the polymer that has the biological properties such as antimicrobial, anti-inflammatory activity, antioxidant for inhibiting oxidation rate of a free radical [26]. Although the XOS and lignin from different biomass have been verified to be applied in the biological filed, limited work was carried out to evaluate if the isolated XOS and solubilized lignin in the prehydrozate by PS-DVB resin possess prebiotics to stimulate the growth of intestinal bacteria and in vitro antioxidant activity.

The overarching purpose of this work was to propose a process to simultaneously separate and recover the solubilized lignin and XOS with yields > 85–90% in dissolving pulp prehydrolyzate by implementing a PS-DVB resin adsorption protocol. Enzymatic hydrolysis with endo-β-1-4-xylanase and subsequent fermentation with *Pichia stipites* were applied to the lignin-free XOS solution to further improve its X2 and X3 contents as well as reduce the concentration of monosaccharides. Structural features of the purified XOS and separated soluble lignin fraction were characterized by 2D-HSQC NMR. Furthermore, free radicals scavenging ability and intestinal activity toward *Bifidobacteria* and *Lactobacillus acidophilus* were evaluated using XOS and soluble lignin to further understand if the constituents in prehydrolyzate can be used as prebiotics and/or antioxidants.

## Methods

### Separating XOS and solubilized lignin dissolved in prehydrolyzate

Moso bamboo, provided by He Qi Cang Bamboo Processing Factory (Fujian, China), was used as raw material to produce the prehydrolyzate. The prehydrolyzate was obtained according to the conventional procedure for dissolving pulp production. Specifically, 1 kg of bamboo was mixed with 10 L water in a 15-L cooking boiler and cooked for 60 min at 180 °C [27]. After prehydrolysis, the liquid (prehydrolyzate) was separated from the cooked solids by cloth bag, and then, the obtained liquid was next centrifuged to remove suspended fine particles. The obtained prehydrolyzate was kept at 4 °C for the following experiments.

PS-DVB resin (Amberlite^®^ XAD16 N) was used to adsorb lignin in order to purify a XOS stream in the prehydrolyzate. Prior to resin usage, sequential washes with water, ethanol, and again water were performed to remove salts and impurities from the resin. After preparation, 80 g of PS-DVB resin was loaded into a Chromaflex glass column (40 × 2 cm). 1 L of prehydrolyzate was passed through the resin column, and the fluid (solubilized sugar solution, termed as S-S solution) was collected. After all the prehydrolyzate eluted, 2 L of deionized water was then eluted to remove soluble carbohydrates remaining within the column. The collected S-S solution and wash solution were mixed and measured for total volume to calculate the recovery yield of sugars. Following separation, 0.5 L of anhydrous ethanol was used to desorb the hydrophobic molecules (solubilized lignin) and the ethanol was collected. Finally, 0.5 L of anhydrous ethanol was again eluted to ensure total adsorbed lignin removal. Two parts solubilized lignin solution (S-L solution) were mixed and measured for total volume to calculate the recovery yield of lignin. Next, ethanol in the S-L solution was evaporated by rotary evaporator and the obtained solids were re-suspended into deionized water and freeze-dried to produce powdered solubilized lignin (S-L) solids.

### Enzymatic hydrolysis and fermentation to purify the XOS from prehydrolyzate

Endo-β-1-4-xylanase (enzyme activity with 17.9 U/mL), provided by Jiangsu Kangwei Biotechnology Co., Ltd. (Yanchen, China), was applied in the enzymatic hydrolysis to improve the contents of xylobiose and xylotriose in XOS from prehydrolyzate. Fifty milliliters of the S-S solution was adjusted to pH of 4.8 and mixed with 5 mL endo-β-1-4-xylanase. This mixture was then kept at 50 °C and agitated using 150 rpm shaking for 12 h, which accorded with optimal condition in our previous work [28]. The enzymatic hydrolysis sugar solution (E-S solution) was obtained for sugar content analysis by high-performance anion exchange chromatography (HPAEC). Finally, E-S solution was evaporated by rotary evaporator to concentrate the sugar for following fermentation.

*Pichia stipites* yeast was used to ferment the monosaccharides in the E-S solution to purify the XOS from prehydrolyzate. The *Pichia stipites* was cultured in a culture solution (containing 30 g/L xylose, 5 g/L peptone, and 3 g/L yeast extraction) at 30 °C and 170 rpm shaking for several batches (24 h per batch). After the *Pichia stipites* yeast grew to the optical density (OD_600_), cells were harvested by centrifugation (3000 rpm for 10 min), washed, and centrifuged three additional times to remove residual sugar and ethanol. *Pichia stipites* inoculum with OD_600_ of 15, 0.24 g/L urea, 0.25 g/L MgSO_4_, 2.5 g/L KH_2_PO_4_, and 0.25 g/L CaCl_2_ were added with the concentrated E-S solution and shaken at 30 °C and 170 rpm for 24 h. After fermentation, the yeast was separated by centrifugation at 8000 rpm for 10 min, and the supernatants were subsequently obtained for sugar content analysis by HPAEC. E-S solution after being fermented by yeast was termed purified sugar solution (P-S solution) and freeze-dried to obtained powdered XOS solids.

### Determination of sugars/lignin in the prehydrolyzate and separated lignin and sugar solutions

Lignin concentrations in prehydrolyzate, S-L solution, and each sugar solution were determined by UV spectrophotometry (*λ* = 280 nm). The lignin concentration was calculated according to the Lambert–Beer law (A = Ɛbc). An aliquot of prehydrolyzate, S-L solution and each sugar solution were taken out for measuring monosaccharide concentrations (glucose and xylose) by high-performance liquid chromatography (HPLC). The HPLC was equipped with a Bio-Rad Aminex HPX-87 H column degasser, pump, and refractive index (RI) detector. 5 mM H_2_SO_4_ was used as the eluent at a flow rate of 0.6 mL/min at the column temperature of 55 °C. Oligosaccharide (xylooligosaccharides and gluco-oligosaccharide) concentrations were determined using a mild acid hydrolysis procedure, in which an aliquot was hydrolyzed by the adding sulfuric acid to reach 4% acid concentration and cooked at 121 °C for 90 min. Quantities of oligosaccharides of xylooligosaccharides and gluco-oligosaccharide in solution were calculated from the differences in their respective monosaccharides contents before and after acid hydrolysis.

The concentrations of XOS with DP of 2–6, including xylobiose (X2), xylotriose (X3), xylotetraose (X4), xylopentaose (X5), and xylohexaose (X6) in S-L solution and each sugar solution were analyzed by HPAEC. The S-L, S-S, E-S, and purified XOS solutions were prepared by dissolving 0.1 g corresponding solid in 10 mL deionized water. The HPAEC was coupled with a pulsed amperometric detector and a PA 200 (2 × 250 mm) column. The separation method to analyze the X2–X5 concentrations was according to the work of Xu et al. [29] and Wang et al. [30] The amount of X2, X3, X4, X5, X6 in S-L, S-S, E-S, and purified XOS was calculated by the concentrations and their percentages were calculated basing on corresponding amounts in 0.1 g solid.

### Characterization of xylooligosaccharides and solubilized lignin by NMR

Two-dimensional heteronuclear single quantum coherence (2D-HSQC) NMR was used to analyze the structural information of the separated XOS and solubilized lignin from prehydrolyzate. Forty milligrams of sample was dissolved in 500 μL DMSO-d_6_ to obtain 2D-HSQC spectra. The acquisition parameters used were 160 transients (scans per block) acquired using 1024 data points in the F2 (^1^H) dimension with an acquisition time of 53 ms and 256 data points in the F1 (^13^C) dimension with an acquisition time of 5.14 ms. Total running time was 18 h. The coupling constant (^1^J_C-H_) of 147 Hz was applied.

### Antioxidant activity of the xylooligosaccharides and solubilized lignin

The antioxidant activities of xylooligosaccharides and solubilized lignin in the prehydrolyzate were assessed by a free radical-scavenging assay using 2,2-diphenyl-1-picryl-hydrazyl (DPPH) radical and hydroxyl radical according to the our previous works [31]. Different concentration solutions of solubilized lignin (0.2–1.2 g/L) and XOS (0.5–3 g/L) were prepared by dissolving the powdered versions of each in distilled water for the scavenging assays.

### Cultivation of intestinal *Bifidobacteria adolescentis* and *Lactobacillus acidophilus*

To activate and inoculate *Bifidobacteria adolescentis*, a medium solution with 50 mL containing of 5 g/L peptone, 5 g/L tryptone, 5 g/L beef extraction, 10 g/L yeast extraction, 10 g/L glucose, 0.08 g/L CaCl_2_, 0.4 g/L NaCl, 0.004 g/L KH_2_PO_4_, 0.4 g/L NaHCO_3_, 0.019 g/L MgSO_4_, 1 g/L l-hemiline, and 1 g/L thioglycolic acid sodium was prepared at pH 7.0. The medium solution for activating and inoculating the *Lactobacillus acidophilus* contained 10 g/L tryptone, 10 g/L beef extraction, 5 g/L yeast extraction, 20 g/L glucose, 5 g/L sodium acetate, 2 g/L ammonium dihydrogen citrate, 2 g/L KH_2_PO_4_, 0.2 g/L MgSO_4_, 0.05 g/L MnSO_4_, 1 g/L l-hemiline, and 1 g/L thioglycolic acid sodium at pH 6.0. The cell powders of *Bifidobacteria adolescentis* and *Lactobacillus acidophilus* were transferred to the 100 mL Erlenmeyer flask and incubated at 30 °C for 36 h in an anaerobic incubator, which contained the inert gases 80% N_2_, 10% CO_2_, and 10% H_2_.

After incubation, 1 mL of *Bifidobacteria adolescentis* and *Lactobacillus acidophilus* inoculum and 19 mL of the corresponding medium solution without glucose were added into the tube. Next, aseptic S-L and XOS powders were added in the tube to reach a substrate concentration of 1% (w/v). Strictly anaerobic conditions and techniques were used during the growth assay of *Bifidobacteria adolescentis* and *Lactobacillus acidophilus* at 37 °C for 72 h. During cultivation, 1 mL of sample was withdrawn at regular intervals (0 h, 6 h, 12 h, 24 h, 48 h, and 72 h) for analysis of bacterial growth and organic acid production. The OD of the *Bifidobacteria adolescentis* and *Lactobacillus acidophilus* in each intervals was measured at 600 nm according to the work of Mäkeläinen et al. [23]. Concentrations of lactic acid, acetic acid, propionate, and butyric acid were measured by the same HPLC procedure used to analyze the monosaccharides.

## Results and discussion

### Evaluation of the process to separate XOS from solubilized lignin in prehydrolyzate

**Fig. 1:**
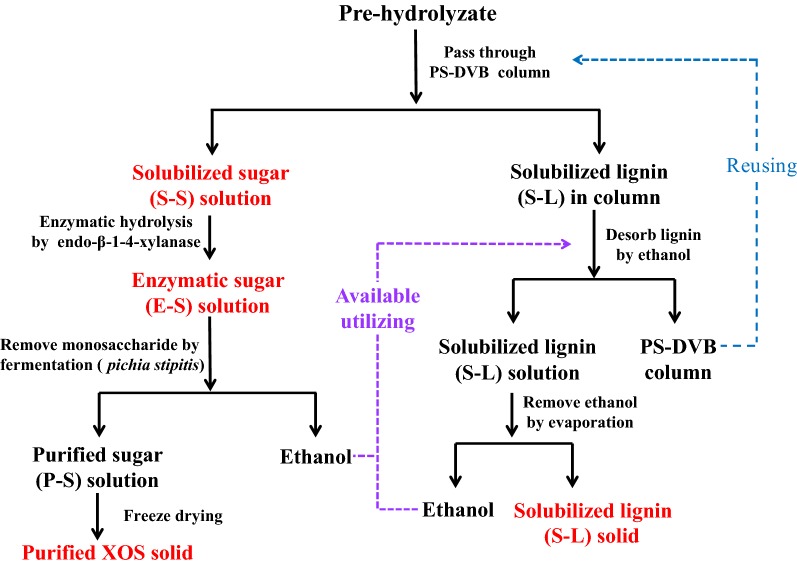
The sustainable and green process to separate XOS and solubilized lignin in prehydrolyzate

Polystyrene divinylbenzene (PS-DVB) is a hydrophobic polymer that has been established to be an effective adsorbent for adsorbing hydrophobic molecules in solution through a combination of *π*–*π*′ and van der Waal interactions. In this work, PS-DVB resin was explored to adsorb the solubilized lignin in order to render a prehydrolyzate enriched in XOS. As shown in Fig. 1, the adsorbed solubilized lignin in resin can be desorbed by ethanol to regenerate the PS-DVB resin for reuse. The re-solubilized lignin solution can then be evaporated to obtain the solubilized lignin (S-L) solid, and the vaporized ethanol is attempted to reuse in the next adsorbing process [22]. The obtained S-L solid (lignin) can be further used to produce lignin-based materials [32, 33].

The passed solubilized sugar (S-S) solution was treated by enzymatic hydrolysis and fermentation process to improve the content of xylobiose (X2) and xylotriose (X3) and remove the monosaccharides (xylose and glucose) in the purified XOS. Compared to selective acid hydrolysis (sulfuric acid and acetic acid) to enhance XOS yield, the biological treatment is regarded as an environmentally friendly process for producing XOS due to discontinued use of acid chemicals and higher XOS yield [9, 11]. For example, an increased XOS yield from 7.1  to 11.2 g/L could be achieved from prehydrolysis liquor by sulfuric acid concentration from 0.1 to 0.3%. A higher XOS yield with 15.7 g/L was obtained under the optimal enzymatic hydrolysis condition [11]. In addition, the fermentation process to degrade the monosaccharides can produce ethanol as a product, which can then be re-circulated to regenerate the PS-DVB resin. Hence, the process to separate high-purity xylooligosaccharides and solubilized lignin in the prehydrolyzate can be regarded as a highly sustainable and green protocol.

### Recovery of components in prehydrolyzate using PS-DVB resin

Quantities of components in the obtained prehydrolyzate, S-L solution, and S-S solution are shown in Table 1. All results are reported in grams based on 1 L prehydrolyzate from 100 g of raw oven-dried Moso bamboo. It could be seen that XOS are the major component in the prehydrolyzate with an amount of 7.4 g, accounting for 42.7% of the original xylan in bamboo. Xylose (1.7 g) was the second most abundant sugar in the prehydrolyzate. The released XOS and xylose are derived from depolymerization of hemicelluloses during the prehydrolysis process [34]. Moreover, a small amount of gluco-oligosaccharide and glucose were released from the bamboo as well. In addition, 3.4 g of solubilized lignin was also quantified in the prehydrolyzate. From all of this, it can be deduced that the prehydrolyzate is a promising source for the production the XOS and lignin due to their considerable amount in the prehydrolyzate.

**Table 1:** The amount of components in prehydrolyzate and separated solution and the separation yields of each components

Component	Amount of components in solution (g)^a^			Recovery yield (%)^b^	Separation yield (%)
	Prehydrolyzate	S-L solution	S-S solution		
Solubilized lignin	3.4 ± 0.1	2.9 ± 0.4	0.4 ± 0.0	97.1 ± 0.5	85.3 ± 0.3^c^
Xylooligosaccharides	7.4 ± 0.6	0.4 ± 0.1	6.9 ± 0.2	98.6 ± 1.1	93.2 ± 0.8^d^
Xylose	1.7 ± 0.2	0.1 ± 0.0	1.4 ± 0.1	94.1 ± 0.1	–
Glucose	0.2 ± 0.0	0.08 ± 0.0	0.1 ± 0.0	90.0 ± 0.0	–
Gluco-oligosaccharide	0.6 ± 0.0	0.07 ± 0.0	0.5 ± 0.2	95.0 ± 0.1	–

^a^Based on 1 L prehydrolyzate from 100 g oven-dried raw bamboo

^b^Amount in S-L solution and S-S solution/amount in prehydrolyzate

^c^Lignin quantity in S-L solution/lignin quantity in prehydrolyzate

^d^Xylooligosaccharides in S-L solution/xylooligosaccharides in prehydrolyzate

In Table 1, it can be seen that 97.1% of solubilized lignin and 98.6% of XOS in the prehydrolyzate can be recovered in the S-L and S-S solutions using PS-DVB resin separation technology (as shown in Fig. 1). Considering the amount of solubilized lignin in S-L solution and XOS in S-S solution, the separation yields of each constituent were 85.3% and 93.2%, respectively. These results reached the desired expectations, which was to simultaneously obtain the solubilized lignin with > 85–90% yields and recover the XOS with > 85–90% yields. Hence, it can be deduced that the PS-DVB resin is an efficient particle to simultaneously separate XOS and solubilized lignin in prehydrolyzate. In theory, no sugars can be found in the desorbed lignin solution and no lignin in the sugar solution, as the PS-DVB resin only engages with hydrophobic solutes (lignin) [21]. Unexpectedly, the obtained S-L and S-S contained the sugars and lignin portion in their composition. As shown in Table 2, the lignin content in S-L was 80.9%, alongside 10.4% XOS, 0.7% xylose, and 2.3% glucose. This means that the isolated S-L was the carbohydrate-enriched lignin. In the work of Narron et al. [22], they also found that the obtained prehydrolyzate-soluble lignin preparation is full of LCC substructures. Meanwhile for S-S solid, it was comprised of 64.0% of XOS, 17.1% of monosaccharides, and 4.5% of lignin. Hence, it is speculated that lignin–carbohydrate complexes (LCC) fragment might inherently exist in S-L and S-S [35], which could provide a reason for why the separation yields of lignin and XOS were not ~ 100%.

**Table 2:** Composition analysis of isolated lignin (S-L), sugars (S-S), and yeast-purified XOS from prehydrolyzate (%)

	S-L	S-S	E-S	Purified XOS
Xylose (X1)	1.7	15.9	22.1	0.5
Xylobiose (X2)	0.7	9.2	23.1	31.8
Xylotriose (X3)	0.6	8.0	19.5	26.9
Xylotetraose (X4)	1.0	6.1	7.1	9.2
Xylopentaose (X5)	2.7	4.6	5.1	7.8
Xylohexaose (X6)	2.2	4.4	4.3	6.2
XOS (DP 2–6)^a^	7.2	32.3	59.1	81.9
XOS (DP > 6)	3.2	31.7	4.1	5.1
Lignin	80.9	4.5	4.9	5.4
Arabinose/arabinan	0.4	1.1	0.7	0.4
Glucose/glucan	2.3	1.2	1.1	0.0
Others	4.3	13.3	8.0	6.7

X1 + XOS + arabinose/araban + glucose/glucan + lignin + others = 100%

^a^XOS (DP 2–6) = X2 + X3 + X4 + X5 + X6

**Fig. 2:**
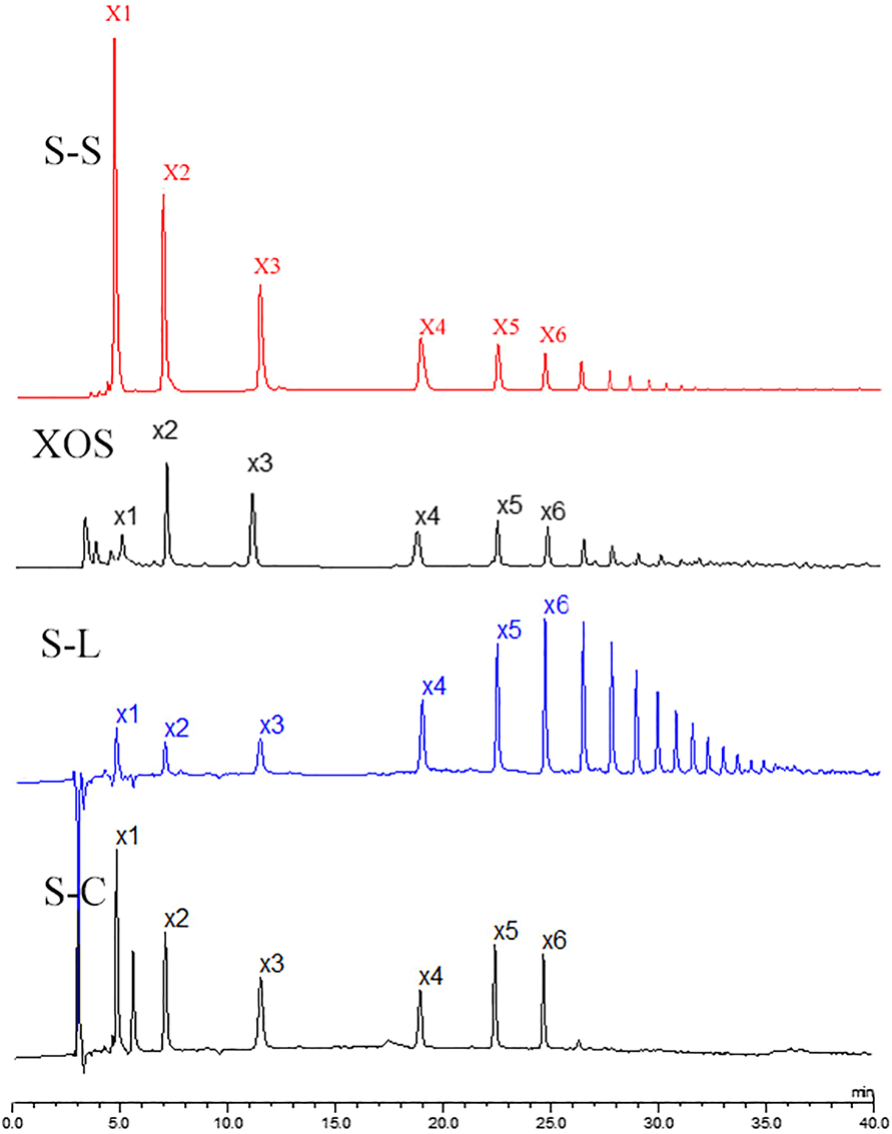
The HAPEC spectra of standard compounds (S-C), S-L, and different sugars

As shown in Table 1, it is found that the xylan and lignin are both the inherent component by composition analysis. In order to explore which kind of xylan is linked with solubilized lignin to form the LCC in S-L and S-S, their compositions were analyzed by HAPEC and results were compared with XOS standard compounds (S-C) of X2–X6. HAPEC spectra of different samples are shown in Fig. 2. It is found that the peaks of X2–X6 were symmetric and well separated in the S-C spectrum, indicating the degree of separation of XOS standards by HAPEC [29]. To our surprise, it is found that S-L contained the structures of X2–X6 similar compared to the standard compounds. This indicated that S-L may contain the fragments of xylan with DP of 2–6. Several peaks were also detected in the S-L spectrum after the peaks of X6, which is attributed to the peak of XOS with DP > 6. Du et al. [36] revealed that xylan with different molecular mass can participate in the LCC formation to be the xylan–lignin complexes. Hence, it is speculated that the xylan with different DP are the inherent component of xylan–lignin complexes in the solubilized lignin. Narron et al. [22] also found that the soluble lignin in prehydrolyzate was found to be heavily degraded but contained a considerable number of lignin–carbohydrate complexes.

### Purifying XOS from prehydrolyzate by sequential enzymatic hydrolysis and fermentation

In Table 2, it was found that the obtained solubilized sugar (S-S) solid from prehydrolyzate was indeed a XOS-containing sample. Specifically, the XOS of DP > 2 were the major components in the S-S, in which the content of XOS (DP 2–6) and XOS (DP > 6) was 32.3% and 31.7%, respectively. In the XOS with DP 2–6, the content of xylobiose (X2), xylotriose (X3) xylotetraose (X4), xylopentaose (X5), and xylohexaose (X6) was 9.2%, 8.0%, 6.1%, 4.6%, and 4.4%, respectively. It is generally considered that the biological functions of XOS are strongly related to their DP, in which the X2 and X3 are favored for stimulating growth of intestinal bifidobacteria [28, 37]. The total amount of X2 and X3 in S-S was just 17.2%. Hence, it is necessary to improve the proportions of X2 and X3 in the XOS obtained from prehydrolyzate in this work.

Huang et al. [38] and Chen et al. [11] elucidated that the acetic acid and sulfuric acid can significantly improve the X2 and X3 yield from the biomass by catalyzing hydrolysis of XOS with DP > 6. However, it is impossible to prevent degradation of XOS into xylose by these acidic means. From the perspective with green process, enzymatic hydrolysis is an attractively green and facile technology that is carried out at much milder conditions that avoid generation of harmful and inhibitory by-products from acid catalysis (hydroxymethylfurfural, furfural, levulinic acid, etc.) [11]. However, the efficiency of enzymatic hydrolysis with xylanase is very sensitive to the increase the yield of XOS with specific DP from the substrate. Hence, enzymatic hydrolysis with endo-β-1-4-xylanase was applied to S-S in order to improve the contents of X2 and X3 in the final product, termed as E-S solid. As shown in Table 2, the enzymatic hydrolysis technology indeed significantly increased the content of X2 and X3 in E-S, with the amount being 42.6% higher than that in S-S (17.2%). In addition, it was found that the amount XOS with DP > 6 decreased from 31.7 to 4.1% after enzymatic hydrolysis. Hence, it can be deducted that the increased amount of X2 and X3 in E-S was mainly attributable to degradation of XOS with DP > 6. The works of Guigon et al. [39] and Huang et al. [28] also found that the enzymatic treatment was a very effective approach for increasing the XOS concentration in the prehydrolyzate, reporting similar results regarding X2 and X3 improvements.

When XOS is considered as the prebiotic for food additives, the presence of xylose is regarded as an impurity whose content should be minimized to less than 1% [40]. This means that further purification of E-S solid to remove xylose is also of great importance to generate valuable XOS products. It has been reported that fermentation technology can be applied to selectively decrease the xylose content in XOS solution by fermenting the xylose into ethanol or other by-products without affecting the concentrations of oligosaccharides. It is also worth considering that the produced ethanol could also be applied to regenerate PS-DVB resin around lignin adsorption, as demonstrated in Fig. 1. With these concepts, fermentation by *Pichia stipites* was applied to purify the E-S because it is capable of converting xylose into ethanol [41]. As shown in Table 2, the content of xylose was significantly reduced from 22.1 (E-S) to 0.5% (purified XOS) after fermentation. Contents of XOS with DP 2–6 correspondingly increased from 59.1 to 81.9%, which satisfies commercial product standards of 80% purity. It should be pointed out that the purified XOS still contained 6.7% other constituent, which might be the ensuing enzymes proteins and mineral salts during purified process. These constituents can successfully be removed by ultrafiltration and electroosmosis technologies. Overall, the results demonstrated that the sequent enzymatic hydrolysis and fermentation process can purify XOS from the prehydrolyzate, accompanying with enriching the contents of X2 and X3 in XOS.

### Structural features of XOS and solubilized lignin by NMR characterization

**Fig. 3:**
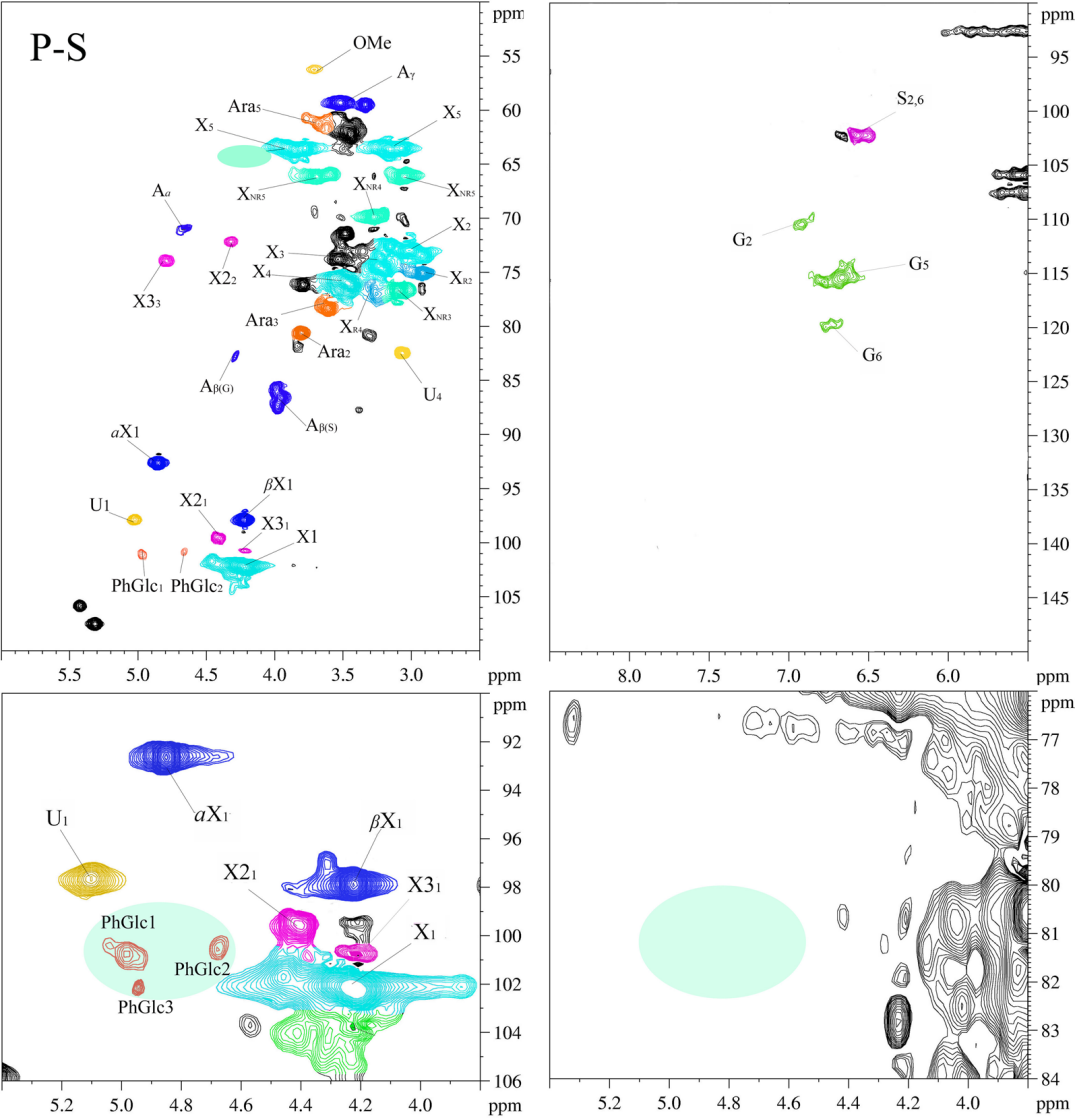

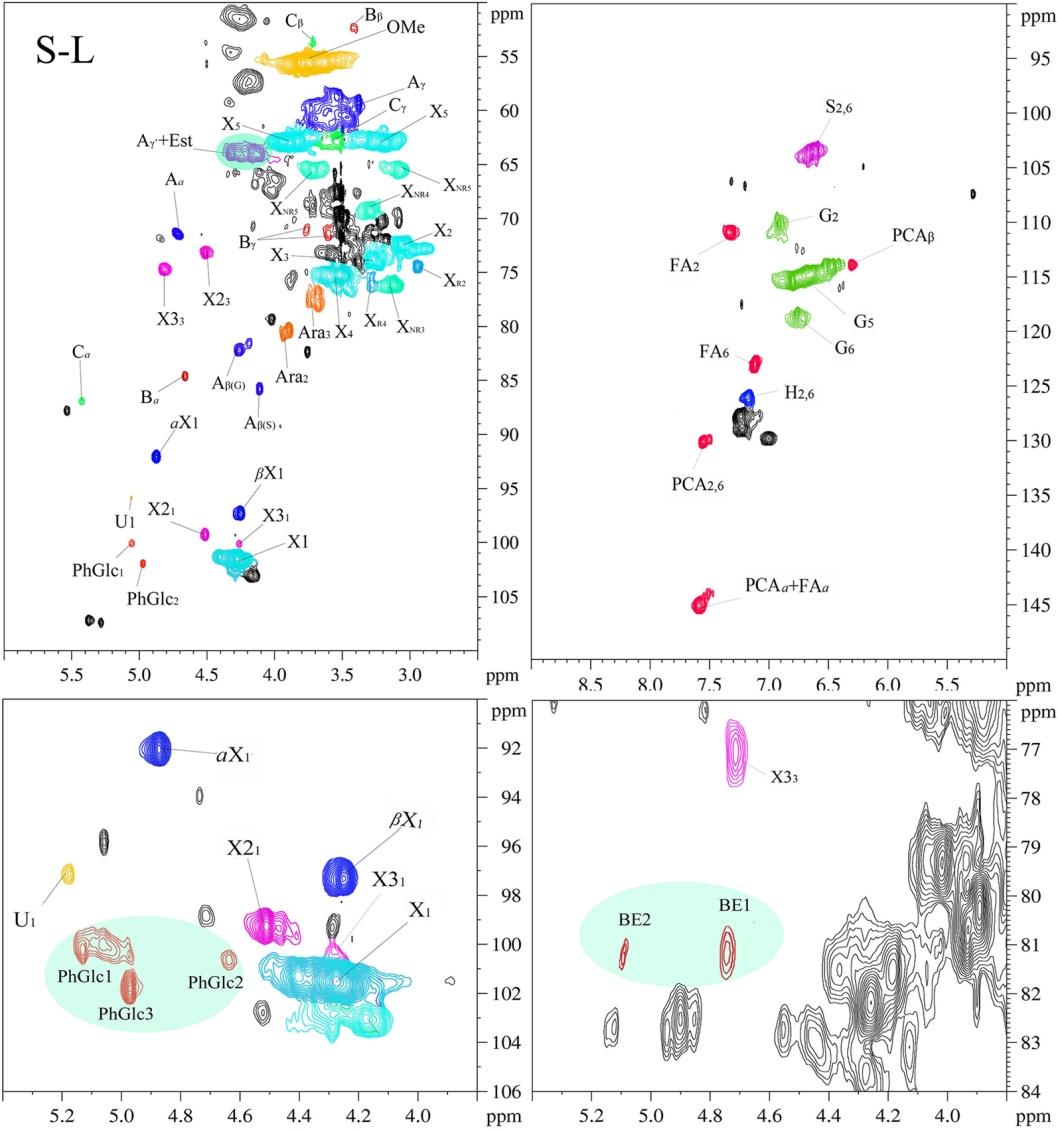
2D-HSQC spectra of S-L and purified XOS

To better understand what are the properties of the components in the prehydrolyzate and why they show characteristics of sugar in the lignin and XOS, the structure features of the purified XOS and solubilized lignin are analyzed by 2D-HSQC NMR spectroscopy technology. The obtained spectra are shown in Fig. 3, and assigned peaks are illustrated in Additional file 1: Table S1 according to published reports [42–44]. The main substructures in the 2D-HSQC spectra are depicted in Additional file 2: Fig. S1.

As shown in Table 2, xylan quantities can be measured in the S-L and XOS, which can also be verified by their correlation signals in the 2D-HSQC spectra. In Fig. 3, the signals for β-(1 → 4)-d-xylopyranoside of internal xylan (X), β-(1 → 4)-d-xylopyranoside with reducing end (X_R_), β-(1 → 4)-d-xylopyranoside with non-reducing end (X_NR_), 2-*O*-acetyl-β-d-xylopyranoside (X2), 3-*O*-acetyl-β-d-xylopyranoside (X3), (1 → 4)-*a*-d-xylopyranoside (*a*X), (1 → 4)-β-d-xylopyranoside (βX), *a*-(1 → 4)-l-arabinofuranoside (Ara), 4-*O*-methyl-*a*-d-GlcUA (U) were found in the XOS spectra, indicating that XOS was derived from 4-*O*-methylglucuronoarabinoxylan [45]. Surprisingly, the S-L spectra also showed the signals of these sugars structures, indicating that the 4-*O*-methylglucuronoarabinoxylan may participate in the forming of LCC in the solubilized lignin in prehydrolyzate. In addition, both the S-L and XOS exhibited dissimilar sugar signal intensities, which suggests that the XOS and S-L contained a mixture of several molecules or DP of xylan, which is in accordance with the results in the HPAEC spectra.

In Fig. 3, it can be found that both the LCC linkages of benzyl ether (BE), phenyl glycoside (PhGlc), and γ-ester (Est) were found in S-L spectra, while only phenyl glycoside (PhGlc) and γ-ester (Est) were found in XOS spectra [31, 36]. There are two types of native benzyl ether LCC linkages in the S-L spectra: (1) benzyl ethers connecting Cα of lignin to carbohydrate primary hydroxyl groups (BE1, *δ*_C_/*δ*_H_ 80–81/4.5–4.7) and (2) benzyl ethers connecting the Cα of lignin to carbohydrate secondary hydroxyl groups (BE2, *δ*_C_/*δ*_H_ 80–81/4.9–5.1). For PhGlc in S-L and XOS spectra, they showed three correlation signals at *δ*_C_/*δ*_H_ 100.1/5.09 (PhGlc1), 100.9/4.63 (PhGlc2), and 101.9/4.92 (PhGlc3). These indicated that there are different kinds of carbohydrate associated with lignin by phenyl glycoside linkage to form LCC in the S-L and XOS [31, 36]. Cγ ester is another LCC linkage that exists between hemicellulosic uronic acid substituents and lignin’s side chain Cγ. The signals of CH_2_-γ in γ-esters were observed in the area of *δ*_C_/*δ*_H_ 65–62/4.5–4.0, which can be overlapped by the signals of γ-acylated β-*O*-4 aryl ethers [22, 46]. The strong presence of LCC linkages in S-L and XOS spectra again demonstrated our previous speculation that the lignin fragments in XOS solid and the XOS fragment in solubilized lignin exist in the form of LCC structures.

In the S-L spectra, the common lignin substructures of β-*O*-4 (A), β-β (B), and β-5 (C) were observed by their unique C_*a*_-H_*a*_ signals at *δ*_C_/*δ*_H_ 71.8/4.86, 84.9/4.69, and 86.8/5.49, respectively. These results indicated that these substructures survive during the hot water prehydrolysis process, which has also been reported in Narron et al [22]. Interestingly, only the β-*O*-4 (A) substructure was found in the XOS spectra, which were identified by all three side-chain signals at *δ*_C_/*δ*_H_ 71.8/4.86 (C_*a*_-H_*a*_), 86.0/4.11 and 86.8/3.99 (C_*β*_-H_*β*_), and 59.6–60.8/3.37–3.72 (C_*γ*_-H_*γ*_). This revealed the residual lignin in the XOS may be linked with carbohydrate only by the β-*O*-4 linkage, indicating the lignin attached to xylan has at least a dimeric form by this inter-lignin linkage. In the aromatic region (*δ*_C_/*δ*_H_ 100–135/5.5–8.5) in S-L and XOS spectra, the correlation signals for syringyl units (S) and guaiacyl units (G) can be observed. The signals at *δ*_C_/*δ*_H_ 111.0/7.01, 114.4/6.73, and 119.0/6.82 were attributed to the C_2_-H_2_, C_5_-H_5_, and C_6_-H_6_ in G units. The C_2,6_-H_2,6_ correlations of S units appeared at *δ*_C_/*δ*_H_ 104.1/6.74. The signals of these units in XOS were much weaker than those from S-L, indicating that only a small amount of lignin existed in the XOS. This is consistent with the compositional analysis results (Table 2). In addition, *p*-coumaric acid (PCA) and ferulic acid (FA) were identified in the S-L spectra, while they cannot be found in the XOS spectra.

### Cultivation of intestinally beneficial bacterias by XOS and solubilized lignin

XOS is a promising prebiotic product because it can selectively promote the growth of beneficial bacterias in the colon, effectively improving human health [23]. In this work, XOS was also found in lesser quantities within the solubilized lignin fraction. To investigate if the biomass-derived products in prehydrolyzate possess the in vitro biological activities for beneficial bacterias, both purified XOS and S-L were applied to cultivate the intestinal *Bifidobacteria adolescentis* and *Lactobacillus acidophilus*.

**Fig. 4:**
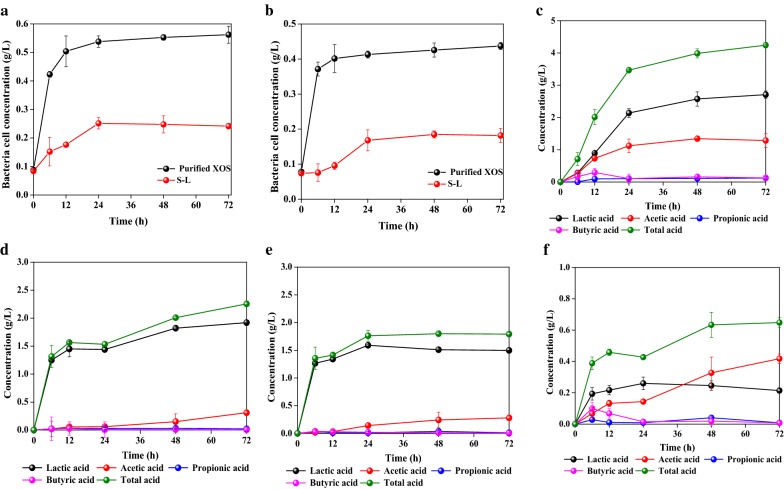
Cultivation of *Bifidobacteria adolescentis* and *Lactobacillus acidophilus* by XOS and solubilized lignin

It is reported that the XOS can be fermented by intestinal bacteria into short-chain fatty acids (SCFA), including lactic acid, acetic acid, propionic acid, and butyric acid [23, 24]. Intestinally produced SCFA have been shown to provide a number of beneficial effects on host health, such as improved bowel function, calcium absorption, lipid metabolism, and a reduction of the risk of colon cancer. Therefore, the extent of stimulated production of SCFA by *Bifidobacteria adolescentis* attributable to XOS and solubilized lignin metabolism is shown in Fig. 4c, d, respectively. Furthermore, SCFA results from *Lactobacillus acidophilus* cultivated by XOS and solubilized lignin are shown in Fig. 4e, f, respectively. It can be seen that each analyzed SCFA (lactic acid, acetic acid, propionic acid, and butyric acid) can be produced during the cultivation of *Bifidobacteria adolescentis* and *Lactobacillus acidophilus* by consuming the XOS and S-L. Across all the produced SCFA, lactic acid was the main product. For example, > 1.5 g/L of lactic acid can be produced from *Bifidobacteria adolescentis* and *Lactobacillus acidophilus* when cultivated with XOS for 24 h. However, the amount of propionic acid and butyric acid was low, with concentrations < 0.1 g/L. Similar SCFA production phenomena were also found from the *Bifidobacteria adolescentis* and *Lactobacillus acidophilus* by consuming the S-L as substrates.

Figure 4c, e demonstrated that XOS and S-L could stimulate the production of SCFA from *Bifidobacteria adolescentis* and *Lactobacillus acidophilus*, but *Bifidobacteria adolescentis* exhibited significantly greater production levels after reaching a final total acid concentration of 3.98 g/L and 2.01 g/L at 48 h. In addition, it was found that the total acid concentration from bacteria consuming the S-L as substrates was lower than XOS. This obviously reveals that the purified XOS preparation provided a greater amount of available substrate for the cultivation of intestinally beneficial bacteria, a result matching the findings from the bacterial growth tests (Fig. 4a). Li et al. [47] also found that the prebiotic stimulated the growth of both *Bifidobacterium* and *Lactobacillus* species, but *Bifidobacterium* exhibited statistically significantly stronger growth stimulation. Overall, the results showed the purified XOS and S-L from prehydrolyzate were capable of providing stimulation to the intestinally beneficial bacteria, demonstrating that the biomass-derived products in prehydrolyzate can be used as viable prebiotics.

### XOS and solubilized lignin antioxidant activities in vitro

**Fig. 5:**
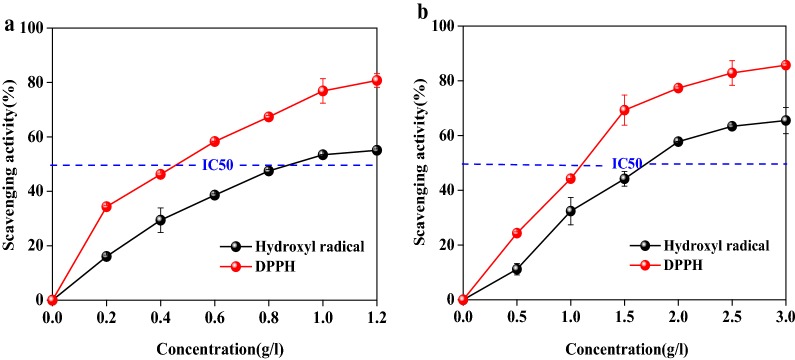
Antioxidant activity of solubilized lignin (**a**) and XOS (**b**) for scavenging DPPH and hydroxyl radical

Free radicals, such as hydroxyl radical and superoxide anion radical, are commonly regarded as one of root for causing many life-threatening diseases. Hence, scavenging these radicals in vivo became a hot topic in relevant medical sciences [48]. To explore whether the components in prehydrolyzate have any additionally beneficial antioxidant capacity, S-L and XOS were analyzed in radical-scavenging assays using DPPH and hydroxyl radicals, which has been considered as the conventional substrates to evaluate the antioxidant capacity [26, 45]. Assay results showed that the antioxidant activities of S-L and XOS were both dose-dependent behavior at concentrations from 0.2–1.2 g/L and 0.5–3 g/L, respectively (Fig. 5). The scavenging activity for DPPH and hydroxyl radical of S-L (Fig. 5a) gradually increased from 34.3 and 16.1% (0.2 g/L) to 80.7 and 55.2% at the concentration of 1.2 g/L, respectively. For XOS (Fig. 5b), the scavenging activity gradually increased to 85.7% for DPPH and 65.5% for hydroxyl radical at 2.5 g/L, after which it remained constant.

IC50 is concentration of antioxidant required to quench 50% of the initial radical, which can be applied to evaluate antioxidant capacity of different substrates. IC50 values of S-L for DPPH and hydroxyl radical scavenging were 0.46 g/L and 0.91 g/L, which were lower than that of XOS with the value of 1.1 g/L and 1.7 g/L. These results indicate that S-L exhibits better performance for scavenging free radicals than XOS. This can be attributed to that the obtained S-L is predominantly lignin, which has abundant content of phenolic and aliphatic hydroxyl groups that benefit for scavenging radicals. Huang et al. [31] reported that the radical-scavenging activities of LCC fractions significantly increased when enhancing the amount of hydroxyl groups in the lignin structure. In addition, the radical-scavenging activity of XOS from prehydrolyzate is comparable to results reported for XOS samples obtained from wood, rice, and sugarcane bagasse [25, 45]. The results obtained from these assays indicated that the isolated solubilized lignin (S-L) and the purified XOS demonstrate a valuable antioxidant capacity that could be usefully applied in foodstuffs as functional ingredients.

## Conclusions

The proposed strategy of sequentially combining hydrophobic resin separation, enzymatic hydrolysis, and fermentation was successfully demonstrated and resulted in simultaneous production of high-quality XOS and solubilized lignin. To further highlight this technique’s sustainability, the separated XOS and lignin were recovered with high yields (85.3% and 93.2%, respectively) and the adsorptive resin can be re-used by ethanol regeneration. The content of xylobiose and xylotriose in the purified XOS product was 58.7%, and the total content of oligosaccharides ranging in DP from 2 to 6 was 81.9%. 2D-HSQC NMR revealed that lignin carbohydrate complexes existed in both XOS and S-L, mainly through covalent linkages between lignin and 4-*O*-methylglucuronoarabinoxylan. In demonstration of each product bioactivity, the DPPH and hydroxyl radical-scavenging behaviors showed both XOS and S-L exhibit the dose-dependent antioxidant activity. Furthermore, XOS stimulated the growth of *Bifidobacteria adolescentis* and *Lactobacillus acidophilus* ~ 600 times, while S-L had a similar effect but to a lesser degree (~ 250 times). This work proves the concept that valuable prebiotic and antioxidant biomass-derived chemicals can be obtained from dissolving pulp prehydrolyzate.

## Additional files

**Additional file 1: Table S1.** Assignment signals of substructure and LCCs linkages in the 2D HSQC spectra of the XOS and S-L preparations.

**Additional file 2: Fig. S1.** Main structures in the XOS and S-L preparations.

## Data Availability

All data generated and analyzed in this study are included in this published article.

## Abbreviations

XOS: xylooligosaccharides
DP: degree of polymerization
PS-DVB: polystyrene divinylbenzene resin
HSQC: heteronuclear single quantum coherence
NMR: nuclear magnetic resonance
S-S: solubilized sugar
S-L: solubilized lignin
E-S: enzymatic hydrolysis sugar
HPLC: high-performance liquid chromatography
HPAEC: high-performance anion exchange chromatography
OD: optical density
P-S: purified sugar
X1: xylose
X2: xylobiose
X3: xylotriose
X4: xylotetraose
X5: xylopentaose
X6: xylohexaose
DPPH: 2-diphenyl-1-picryl-hydrazyl
LCC: lignin–carbohydrate complexes
S-C: standard compounds
BE: benzyl ether
PhGlc: phenyl glycoside
Est: γ-ester
SCFA: short-chain fatty acids
IC50: concentration of antioxidant to quench 50% of the initial radical
