# Supplementary material for: A sustainable process for procuring biologically active fractions of high-purity xylooligosaccharides and water-soluble lignin from Moso bamboo prehydrolyzate
Source: Biotechnol Biofuels. 2019 Jul 29;12:189. doi: 10.1186/s13068-019-1527-3 (PMC6661736; doi:10.1186/s13068-019-1527-3)
Supplement: Supplementary file 1 — Additional file 1: Table S1. Assignment signals of substructure and LCCs linkages in the 2D HSQC spectra of the XOS and S-L preparations. [file 13068_2019_1527_MOESM1_ESM.docx]

Table S1 Assignment signals of substructure and LCCs linkages in the 2D HSQC spectra of the XOS and S-L preparations

| Labels | δ_C_/δ_H_ | Assignment |
| --- | --- | --- |
| Lignin structure | | |
| C*_β_* | 53.1/3.49 | C*_β_*-H*_β_* in phenylcoumaran substructures (C) |
| B*_β_* | 53.7/3.05 | C*_β_*-H*_β_* in resinol substructures (B) |
| -OCH_3_ | 55.9/3.73 | C-H in methoxyls |
| A*_γ_* | 59.6-60.8/3.37-3.72 | C*_γ_*-H*_γ_* in *β*-O-4 substructures(A) |
| A'*_γ_* | 63.6/4.36 | C*_γ_*-H*_γ_* in *γ*-acylated *β*-O-4 substructures (A') |
| B*_γ_* | 71.3/4.18,3.82 | C*_γ_*-H*_γ_* in resinol substructures (B) |
| A*_β_*_（G/H）_ | 83.9/4.30 | C*_β_*-H*_β_* in *β*-O-4 substructures linked to a G unit (A) |
| B*_a_* | 84.9/4.69 | C*_a_*-H*_a_* in resinol substructures (B) |
| A*_β_*_（S）_ | 86.0/4.11 | C*_β_*-H*_β_* in *β*-O-4 substructures linked to a S unit (A) |
| C*_a_* | 86.8/5.49 | C*_a_*-H*_a_* in phenylcoumaran substructures (C) |
| S_2,6_ | 104.1/6.74 | C_2,6_-H_2,6_ in etherified syringyl units (S) |
| G_2_ | 111.0/7.01 | C_2_-H_2_ in guaiacyl units (G) |
| FA_2_ | 111.1/7.34 | C_2_-H_2_ in ferulate (FA) |
| PCA*_β_* | 113.8/6.29 | C_8_-H_8_ in *p*-coumarate (PCA) |
| G_5_ | 114.4/6.73 | C_5_-H_5_ in guaiacyl units (G) |
| PCA_3,5_ | 116.2/6.77 | C_3_-H_3_ and C_5_-H_5_ in *p*-coumarate (PCA) |
| G_6_ | 119.0/6.82 | C_6_-H_6_ in guaiacyl units (G) |
| FA_6_ | 123.1/7.19 | C_6_-H_6_ in ferulate (FA) |
| H_2,6_ | 127.8/7.22 | C_2,6_-H_2,6_ in *p*-hydroxyphenyl units (H) |
| PCA_2,6_ | 130.1/7.48 | C_2,6_-H_2,6_ in *p*-hydroxyphenyl units (H) |
| PCA*_a_*, FA*_a_* | 144.7/7.46 | C*_a_*-H*_a_* in *p*-coumarate (PCA) and ferulate (FA) |
| Associated carbohydrate and LCCs linkages | | |
| Ara_5_ | 61.9/3.52 | C_5_-H_5_ in *a*-(1→4)-L-arabinofuranoside |
| X_5_ | 62.6/3.40 | C_5_-H_5_ in *β-*(1→4)-D-xylopyranoside |
| X_NR5_ | 65.5/3.01,3.65 | C_5_-H_5_ in *β-*(1→4)-D-xylopyranoside with non-ducing end |
| X_NR4_ | 69.5/3.24 | C_4_-H_4_ in *β-*(1→4)-D-xylopyranoside with non-ducing end |
| X_2_ | 72.5/3.02 | C_2_-H_2_ in *β*-(1→4)-D-xylopyranoside |
| X2_2_ | 73.2/4.49 | C_2_-H_2_ in 2-O-acetyl-*β*-D-xylopyranoside |
| X_3_ | 73.7/3.22 | C_3_-H_3_ in *β*-(1→4)-D-xylopyranoside |
| X3_3_ | 74.7/4.80 | C_3_-H_3_ in 3-O-acetyl-*β*-D-xylopyranoside |
| X_4_ | 75.4/3.60 | C_4_-H_4_ in *β*-(1→4)-D-xylopyranoside |
| Ara_3_ | 77.1/3.72 | C_3_-H_3_ in *a*-(1→4)-L-arabinofuranoside |
| U_4_ | 81.1/3.11 | C_4_-H_4_ in 4-O-methyl-α-D-GlcUA |
| Ara_2_ | 81.6/3.89 | C_3_-H_3_ in *a*-(1→4)-L-arabinofuranoside |
| *a*X_1_ | 92.0/4.88 | C_1_-H_1_ in (1→4)-*a*-D-xylopyranoside |
| U_1_ | 97.2/5.18 | C_1_-H_1_ in 4-O-methyl-α-D-GlcUA |
| *β*X_1_ | 97.4/4.26 | C_1_-H_1_ in (1→4)-*β*-D-xylopyranoside |
| X2_1_ | 99.4/4.52 | C_1_-H_1_ in 2-O-acetyl-*β*-D-xylopyranoside |
| X3_1_ | 101.6/4.32 | C_1_-H_1_ in 3-O-acetyl-*β*-D-xylopyranoside |
| X_1_ | 103.2/4.21 | C_1_-H_1_ in *β*-(1→4)-D-xylopyranoside |
| Est+A'γ | 65-62/4.5-4.0 | γ-ester and A'γ in LCC |
| BE_1_ | 81.4/4.64 | Benzyl ether (in primary OH of carbohydrate) in LCC |
| BE_2_ | 81.2/5.06 | Benzyl ether (in secondary OH of carbohydrate) in LCC |
| PhGlc1 | 100.1/5.09 | Phenyl glycoside-1 in LCC |
| PhGlc2 | 100.9/4.63 | Phenyl glycoside-2 in LCC |
| PhGlc3 | 101.9/4.92 | Phenyl glycoside-3 in LCC |
