# Supplementary material for: A sustainable process for procuring biologically active fractions of high-purity xylooligosaccharides and water-soluble lignin from Moso bamboo prehydrolyzate
Source: Biotechnol Biofuels. 2019 Jul 29;12:189. doi: 10.1186/s13068-019-1527-3 (PMC6661736; doi:10.1186/s13068-019-1527-3)
Supplement: Supplementary file 2 — Additional file 2: Fig. S1. Main structures in the XOS and S-L preparations. [file 13068_2019_1527_MOESM2_ESM.doc]

Figure S1 Main structures in the XOS and S-L preparations
